# Supplementary material for: Hyperuricaemia Does Not Interfere with Aortopathy in a Murine Model of Marfan Syndrome
Source: Int J Mol Sci. 2023 Jul 10;24(14):11293. doi: 10.3390/ijms241411293 (PMC10379183; doi:10.3390/ijms241411293)
Supplement: Supplementary file 1 [file ijms-24-11293-s001.zip › ijms-2441887-supplementary.pdf]

SUPPLEMENTARY MATERIAL

Hyperuricaemia Does Not Interfere with Aortopathy in a Murine Model of Marfan Syndrome

Isaac Rodríguez-Rovira [1], Angela López-Sainz [2], Maria Encarnación Palomo-Buitrago [1], Belen Pérez [3], Francesc Jiménez-Altayó [3], Victoria Campuzano [1,4] and Gustavo Egea [1,5,*]

1 Department of Biomedical Sciences, School of Medicine and Health Sciences, University of Barcelona, 08036 Barcelona, Spain
2 Department of Cardiology, Hospital Clínic de Barcelona, IDIBAPS, 08036 Barcelona, Spain
3 Department of Pharmacology, School of Medicine, Autonomous University of Barcelona, Bellaterra, 08192 Barcelona, Spain
4 Centro de Investigación Biomédica en Red de Enfermedades Raras (CIBERER), ISCIII, 28029 Madrid, Spain
5 UZA/UA Center of Medical Genetics, University of Antwerp, 2650 Edegem, Belgium
* Correspondence: gegea@ub.edu

SUPPLEMENTARY FIGURE LEGENDS

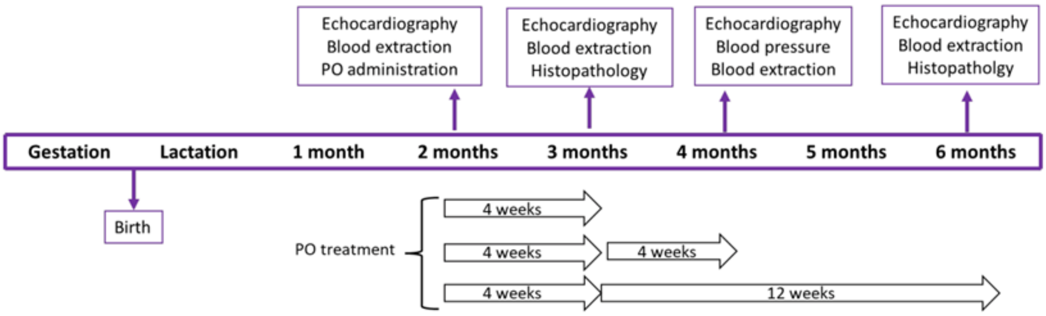

Supplementary Figure S1. Representative scheme of the experimental protocols for allopurinol treatments. PO: potassium oxonate. PO was administered to WT and MFS mice for four weeks. Some animals were euthanized and other continued receiving PO for additional 4 weeks or 12 weeks for a total time of 8 and 16 weeks, respectively.

Received: 24 May 2023
Revised: 30 June 2023
Accepted: 7 July 2023
Published: 10 July 2023

Copyright: © 2023 by the authors. Submitted for possible open access publication under the terms and conditions of the Creative Commons Attribution (CC BY) license (https://creativecommons.org/licenses/by/4.0/).

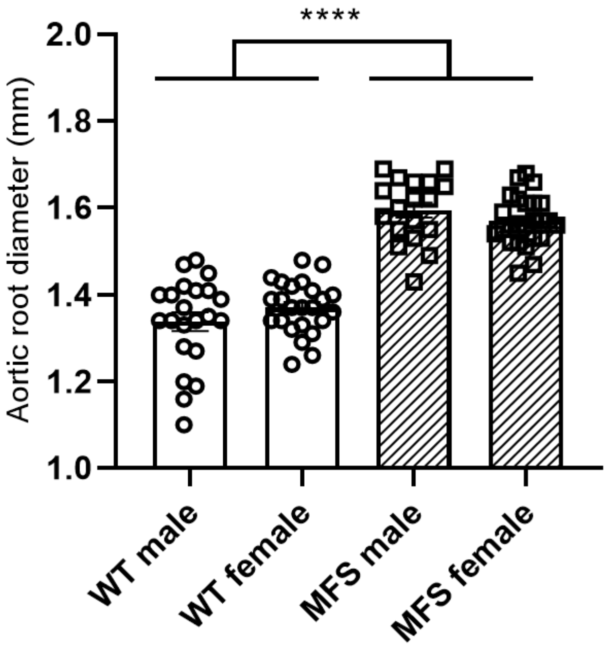

Supplementary Figure S2. Aortic root diameter in WT and MFS mice at the start point of the study (two months old). The aortic root diameter of male and female WT and MFS mice was measured with 2D echocardiography. Results are the mean ± SEM. Statistical test: Two-way ANOVA. ****p ≤0.0001.

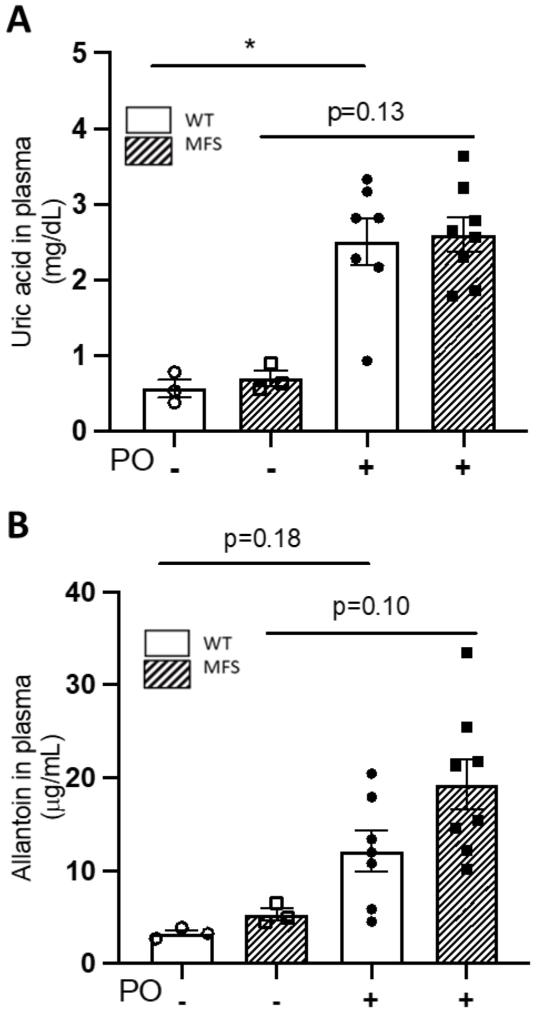

Supplementary Figure S3. Uric acid and allantoin plasma levels following eight weeks of potassium oxonate treatment. Three-month-old WT and MFS mice (treated with PO for four weeks) continued receiving PO treatment for four weeks more (total of eight weeks of PO treatment; four-month-old mice) and their uric acid (A) and allantoin (B) plasma levels were determined. Results are the mean ± SEM. Statistical tests: Kruskal–Wallis, Dunn's post hoc. *p ≤0.05

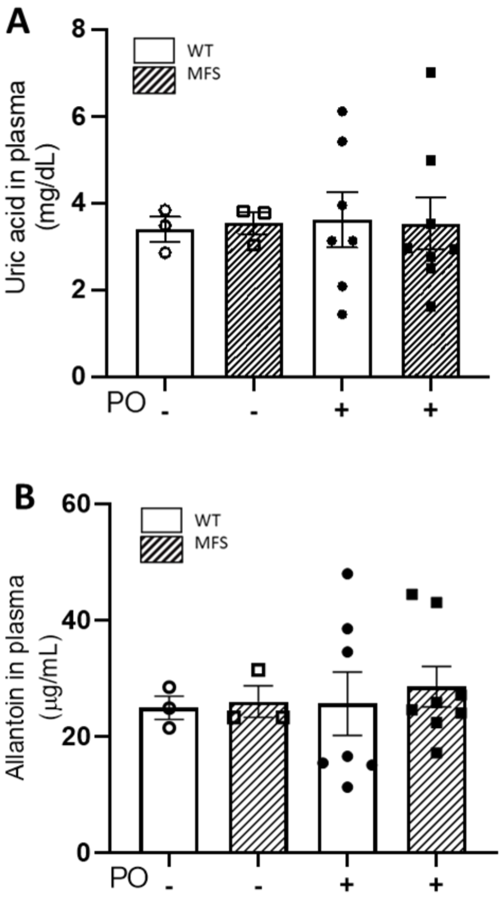

Supplementary Figure S4. Uric acid and allantoin plasma levels following 16 weeks of potassium oxonate treatment. Male WT and MFS mice treated for eight weeks with potassium oxonate (PO) were treated for an additional eight weeks (16 weeks of PO treatment; six-month-old mice); uric acid (**A**) and allantoin (**B**) plasma levels were determined. Results are the mean ± SEM. Statistical test: Kruskal–Wallis.

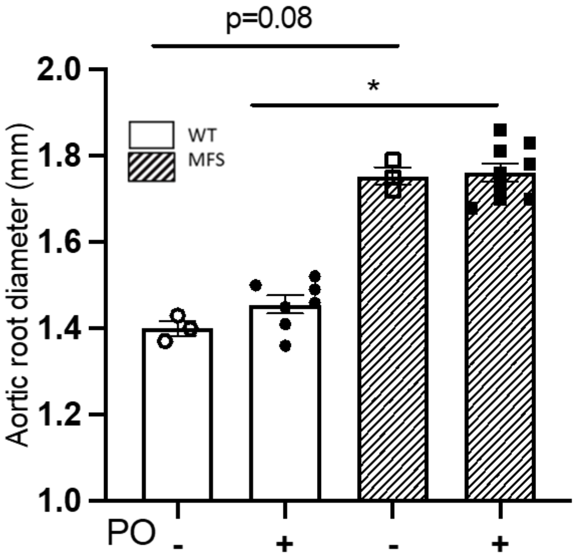

Supplementary Figure S5. Aortic root diameter in WT and MFS mice following eight weeks of potassium oxonate treatment. Male WT and MFS mice treated for four weeks with potassium oxonate (PO) were treated for an additional four weeks (eight weeks of PO treatment; four-month-old mice) and the aortic root diameter was measured again with 2D echocardiography. Results are the mean ± SEM. Statistical tests: Kruskal–Wallis, Dunn's post hoc. *p ≤0.05.

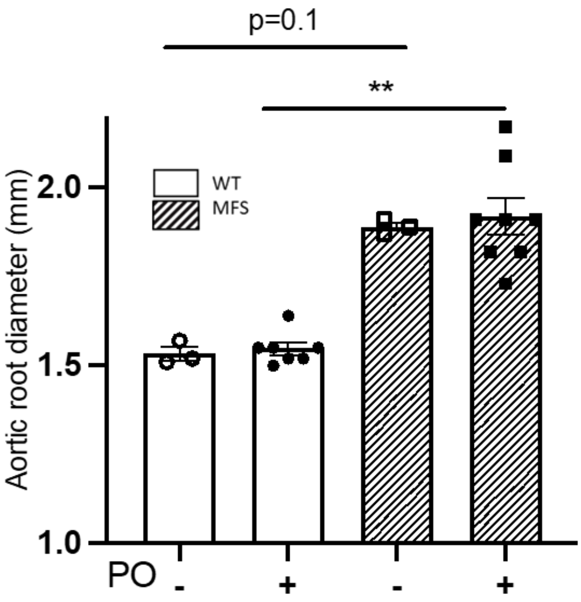

Supplementary Figure S6. Aortic root diameter in WT and MFS mice following 16 weeks of potassium oxonate treatment. Male WT and MFS mice treated for eight weeks with potassium oxonate (PO) were treated for an additional eight weeks (16 weeks of PO treatment; six-month-old mice) and the aortic root diameter was measured again with 2D echocardiography. Results are the mean ± SEM. Statistical tests: Kruskal–Wallis, Dunn's post hoc. ***p* ≤0.01.

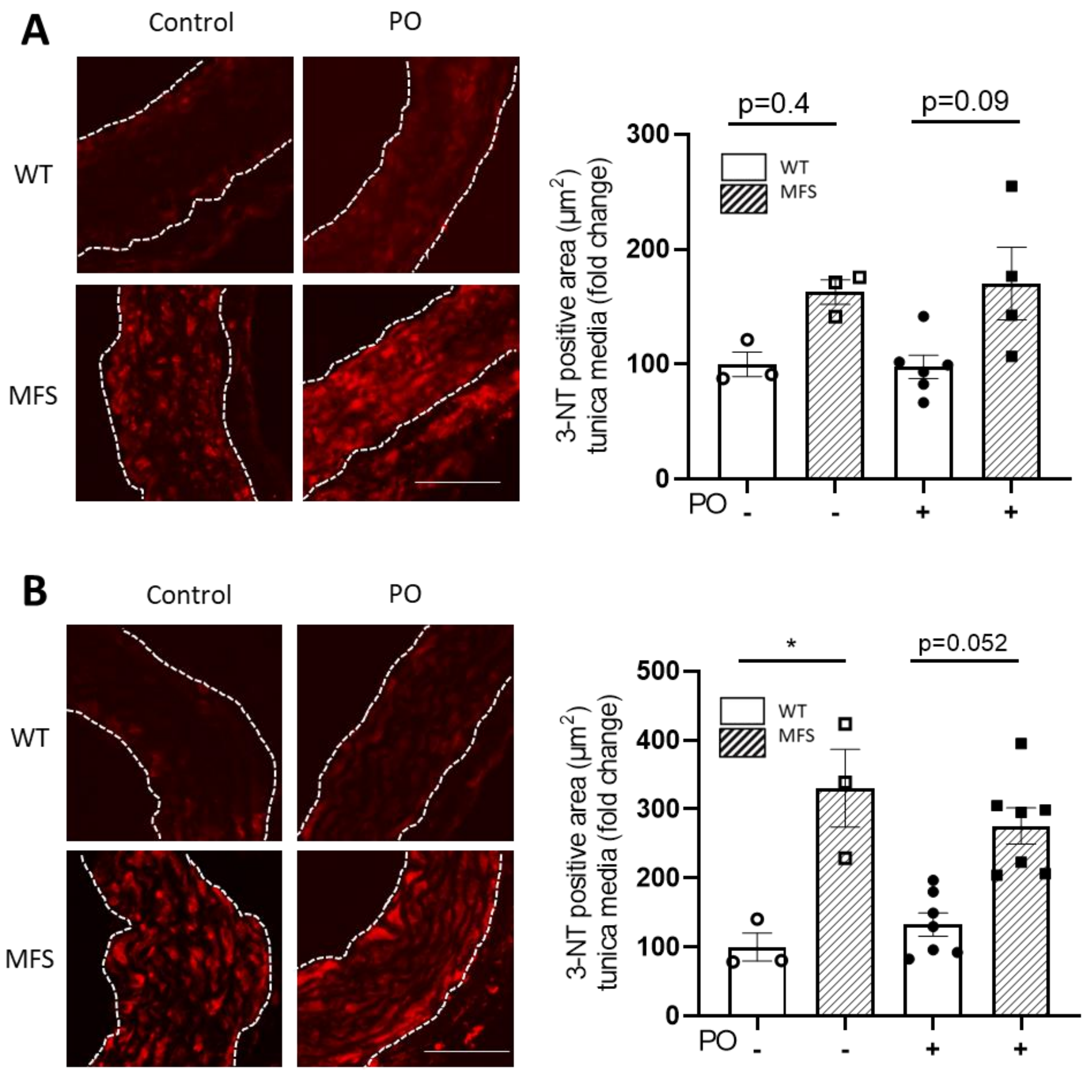

Supplementary Figure S7. **3′-nitrotyrosine levels in the aortic media of WT and MFS mice treated with potassium oxonate.** Representative images and quantitative analysis of 3-nitrotyrosine (3-NT) levels in the aortic tunica media of WT and MFS mice treated with potassium oxonate (PO) for 4 weeks **(A)** and 16 weeks **(B)** (mice aged 3 and 6 months of age, respectively). The tunica media is delineated with dashed white lines. Bar, 100 μm. Statistical tests: Kruskal–Wallis and Dunn's multiple comparison tests. *$p \leq 0.05$.

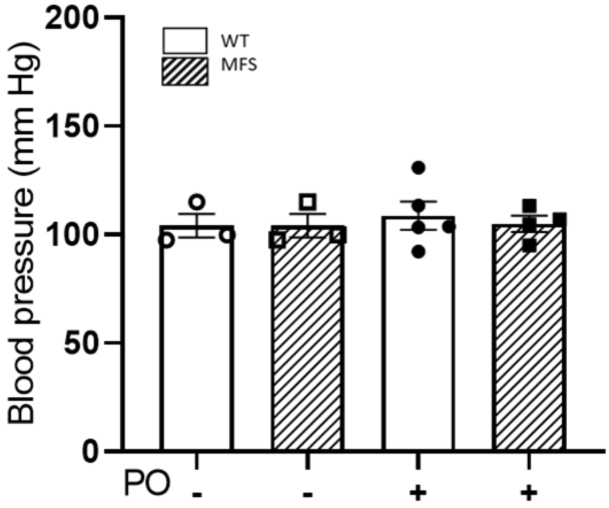

Supplementary Figure S8. Systolic blood pressure in WT and MFS treated with potassium oxonate. Systolic blood pressure measurements in four-month-old WT and MFS mice treated with potassium oxonate (PO; eight weeks). Results are the mean ± SEM. Statistical test: Kruskal–Wallis with Dunn's multiple comparison test.
